# Engram Size Varies with Learning and Reflects Memory Content and Precision

**DOI:** 10.1523/JNEUROSCI.2786-20.2021

**Published:** 2021-05-05

**Authors:** Jessica Leake, Raphael Zinn, Laura H. Corbit, Michael S. Fanselow, Bryce Vissel

**Affiliations:** ^1^Centre for Neuroscience and Regenerative Medicine, Faculty of Science, University of Technology Sydney, Sydney, New South Wales 2007, Australia; ^2^St Vincent's Centre for Applied Medical Research, Sydney, New South Wales 2011, Australia; ^3^Department of Psychology, University of Toronto, Toronto, Ontario M5S 1A1, Canada; ^4^Staglin Center for Brain and Behavioral Health, University of California Los Angeles, Los Angeles, California 90095; ^5^Department of Psychology, University of California Los Angeles, Los Angeles, California 90095; ^6^Department of Psychiatry and Biobehavioral Sciences, University of California Los Angeles, Los Angeles, California 90095

**Keywords:** c-fos, context, engram, fear conditioning, hippocampus, memory

## Abstract

Memories are rarely acquired under ideal conditions, rendering them vulnerable to profound omissions, errors, and ambiguities. Consistent with this, recent work using context fear conditioning has shown that memories formed after inadequate learning time display a variety of maladaptive properties, including overgeneralization to similar contexts. However, the neuronal basis of such poor learning and memory imprecision remains unknown. Using c-fos to track neuronal activity in male mice, we examined how these learning-dependent changes in context fear memory precision are encoded in hippocampal ensembles. We found that the total number of c-fos-encoding cells did not correspond with learning history but instead more closely reflected the length of the session immediately preceding c-fos measurement. However, using a c-fos-driven tagging method (*TRAP2* mouse line), we found that the degree of learning and memory specificity corresponded with neuronal activity in a subset of dentate gyrus cells that were active during both learning and recall. Comprehensive memories acquired after longer learning intervals were associated with more double-labeled cells. These were preferentially reactivated in the conditioning context compared with a similar context, paralleling behavioral discrimination. Conversely, impoverished memories acquired after shorter learning intervals were associated with fewer double-labeled cells. These were reactivated equally in both contexts, corresponding with overgeneralization. Together, these findings provide two surprising conclusions. First, engram size varies with learning. Second, larger engrams support better neuronal and behavioral discrimination. These findings are incorporated into a model that describes how neuronal activity is influenced by previous learning and present experience, thus driving behavior.

**SIGNIFICANCE STATEMENT** Memories are not always formed under ideal circumstances. This is especially true in traumatic situations, such as car accidents, where individuals have insufficient time to process what happened around them. Such memories have the potential to overgeneralize to irrelevant situations, producing inappropriate fear and contributing to disorders, such as post-traumatic stress disorder. However, it is unknown how such poorly formed fear memories are encoded within the brain. We find that restricting learning time results in fear memories that are encoded by fewer hippocampal cells. Moreover, these fewer cells are inappropriately reactivated in both dangerous and safe contexts. These findings suggest that fear memories formed at brief periods overgeneralize because they lack the detail-rich information necessary to support neuronal discrimination.

## Introduction

Developing a comprehensive cognitive map of an environmental context takes time. This is because contexts are composed of many disparate features, only a fraction of which can be attended to at any moment. To encode such multimodal stimuli into memory, animals must sample their features and integrate them into unified representations within hippocampal networks (Fanselow, 1986, 2000; Rudy and O'Reilly, 2001). This process commences rapidly, as indicated by increased immediate early gene (IEG) expression and the emergence of place fields within seconds of entering a novel context (Pevzner et al., 2012). Yet once initiated, the same process continues for many minutes, with progressive increases in hippocampal IEGs and full stabilization of hippocampal place fields only after several minutes of context exploration (Frank et al., 2004; Leutgeb et al., 2004; Leake et al., 2017; Colon and Poulos, 2020). This extended period of hippocampal activity suggests that encoding the entire context may take considerably longer than developing an initial representation. Thus, interrupting the learning process could result in memories impoverished in contextual detail.

Recent research (Zinn et al., 2020) has shown that this rapid initiation and delayed completion of contextual learning can have profound adaptive consequences. Using contextual fear conditioning in mice, we demonstrated that the precision of contextual fear memory is critically dependent on the time animals spend in the context before shock (placement shock interval [PSI]). Animals conditioned at longer PSIs were able to differentiate between the conditioning context and a similar context. However, as the PSI was shortened, fear became increasingly generalized and resistant to extinction. These observations are potentially interesting because overgeneralized and persistent fear is characteristic of post-traumatic stress disorder and other psychiatric disorders (Dunsmoor and Paz, 2015). This suggests that incomplete contextual encoding could be one mechanism through which maladaptive fear arises (Jacobs and Nadel, 1985; Brewin et al., 2010; Brewin, 2014).

Given the potential implications of forming incomplete memories, the goal of the present study was to investigate how time-dependent changes in contextual memory precision are neurally encoded within hippocampal networks. Theoretical models (Treves and Rolls, 1994; Krasne et al., 2015) and empirical research (Liu et al., 2012; Denny et al., 2014; Ryan et al., 2015) have demonstrated that memories for specific contexts are encoded within sparse hippocampal cell populations. When reexposed to the same context, features of that environment trigger reactivation of the original cellular ensemble, resulting in memory recall. However, when exposed to a different environment, a largely nonoverlapping cell population is activated and differentiation occurs (Chawla et al., 2005; Deng et al., 2013; Yokoyama and Matsuo, 2016). Here we examined how the extent of initial contextual learning affects these processes. We predicted that PSI would alter the number of cells activated and incorporated into the memory during learning and retrieval. At shorter PSIs, animals would have less time for environmental sampling, resulting in acquisition of fewer contextual details and recruitment of fewer cells into the memory trace. This in turn would influence the degree to which the same cells would be reactivated across contexts.

To test these possibilities, mice were conditioned at a range of PSIs and c-fos expression was used an indicator of context-encoding related activity within hippocampal ensembles. We began by examining the effect of PSI on the total number of c-fos-expressing cells active after memory acquisition or testing. We then used the c-fos-driven transgenic *TRAP2* mouse line (DeNardo et al., 2019) to tag cells that were active during encoding at different PSIs. Our results reveal that PSI-dependent changes in memory precision correspond with cellular reactivation in a subset of c-fos-expressing cells. In contrast, the total number of c-fos-expressing cells more closely corresponds with session duration. Together, these findings provide insights into the neural substrates of memory precision and the regulation of hippocampal activity in response to previous and ongoing experience.

## Materials and Methods

### 

#### Subjects

Male C57BL6/J mice were obtained from Australian BioResources. Fos^2A-iCreER/+^ (*TRAP2*) mice were obtained from The Jackson Laboratory (Jax #030323) and crossed with ROSA26-CAG-stop-flox-tdTomato (Ai14) mice (Jax #007914) to produce double transgenic mice that were used in experiments. Transgenic experimental mice were heterozygous for the Fos^2A-iCreER/+^ gene and homozygous for the reporter gene.

All mice were 8-12 weeks old at the beginning of experiments. Mice were housed in groups of 2-4 in plastic cages (32 cm × 27 cm × 26 cm) in a temperature-controlled environment (25°C) on a 12 h light-dark cycle (lights on at 0700). All experiments took place during the light phase of the cycle. Food and water were available *ad libitum*. All procedures were approved by the ethics committee at the Garvan Institute of Medical Research and in accordance with the National Health and Medical Research Council animal experimentation guidelines and the Australian Code of Practice for the Care and Use of Animals for Scientific Purposes (2013).

#### Contextual fear conditioning

##### Apparatus

Experiments were conducted in standard fear conditioning chambers (32 cm × 27 cm × 26 cm, Med Associates) connected to a computer installed with FreezeFrame2 (Actimetrics) software. Each chamber consisted of aluminum side walls and clear Plexiglas ceiling, front and back walls. The floor was composed of 36 stainless-steel bars, set 8 mm apart with a waste tray below. The chambers served as both Context A and Context B. To produce Context A, the chambers were wiped down with 80% ethanol and the trays beneath were scented with aniseed essence. To produce Context B, a square white plastic sheet was placed over the grid floor and a longer length of white plastic was extended over the top of square, forming an arch. The chambers were wiped down with 80% isopropanol, which also scented the chambers. Both contexts were illuminated by a houselight, and the room was under full fluorescent lighting. The unconditional stimulus consisted of a 2 s 1 mA footshock, delivered through the grid floor. Mouse behavior was recorded using video cameras positioned in front of the conditioning chambers.

##### Procedure

Mice were habituated to handling procedures at least 3 times before beginning behavioral experiments. For the 4-OHT labeling experiments, mice were habituated to the intraparietal injections once a day for 7 d before beginning experiments.

On conditioning day, mice were transported from the holding room to the fear conditioning room in their home cages and placed in the fear conditioning chamber for various periods of time before footshock. Mice remained in the chamber for a further 30 s before being returned to their home cage. For behavioral testing, mice were returned to either the conditioning context (Context A) or the alternative context (Context B) 24 h after conditioning and allowed to explore the chamber for up to 30 min without shock, as per Results. For the targeted labeling experiments, mice received a single intraparietal injection of 4-OHT immediately after removal from the fear conditioning chamber, before being returned to their home cage. Mice were tested 7 d after conditioning, to allow sufficient time for expression of the transgene.

##### Data analysis

Freezing for each mouse was assessed by an observer blind to the experimental condition. Freezing was defined as immobility except that required for breathing (Bolles, 1970; Fanselow and Bolles, 1979). Freezing was measured using a time sampling procedure in which the mouse was scored as either freezing or not freezing every 4 s. The number of samples scored as freezing was divided by the total number of samples to yield a percentage.

#### Drug preparation

4-Hydroxytamoxifen (4-OHT) was dissolved in 100% ethanol at a concentration of 20 mg/ml by vortexing for 5 min. Once completely dissolved, the solution was mixed with sunflower oil at a concentration of 10 mg/ml by shaking for 15 min. The ethanol was then evaporated by vacuum centrifugation. The final solution was placed in a water bath at 37°C, protected from light, until injection. Mice were injected intraparietally at a concentration of 100 mg/kg.

#### Immunohistochemistry

Sixty minutes after behavioral training or testing, mice were anesthetized with ketamine (8.7 mg/ml) and xylazine (2 mg/ml) and perfused transcardially with ice-cold saline followed by 4% PFA in 1 × PBS. Brains were removed and postfixed for 24 h in the same solution before being transferred to 30% sucrose solution for cryoprotection. Following 72 h in sucrose solution, brains were blocked in OCT and frozen at −80°C until further use. Coronal sections of 40 μm were cut throughout the hippocampal region, with a sectioning interval of 6.

For c-fos immunohistochemistry, free-floating sections were first rinsed 3 times in 1 × PBS and then blocked with 5% BSA with 0.3% Tween 20 in 1 × PBS for 1 h at room temperature. Sections were then incubated in anti-c-fos primary antibody diluted in blocking solution for 72 h at 4°C. Sections were then washed 3 times in 1 × PBS and incubated overnight at 4°C in goat anti-rabbit AlexaFluor-488-conjugated secondary antibody (1: 250, Invitrogen, catalog #A11008). The following day, sections were again washed 3 times in 1 × PBS and counterstained with DAPI (Invitrogen). Finally, sections were washed 3 times in 1 × PBS, mounted onto glass slides, and coverslipped with 50% glycerol. Initial experiments (see Figs. 2, 3*B-D*) were conducted using Santa Cruz Biotechnology anti-c-fos primary antibody (1:500, catalog #sc-52, RRID:AB_2106783). Because of the discontinuation of this product, later experiments (see Figs. 3*E-G*, 4, 5) were conducted using Millipore anti-c-fos primary antibody (1:1000, catalog #ABE457, RRID:AB_2631318).

#### Image acquisition

Fluorescent confocal micrographs were captured using a Leica Microsystems DMI6000 inverted laser scanning confocal microscope with the aid of the Leica Application Suite X software platform. For individual cell counts, single images were captured at a *z* depth of 10 μm to avoid cutting artifacts at the edges of the section. For colabeled cell quantification, 10 μm *Z* stacks were acquired through the thickness of the tissue. Quantification of c-fos expression was restricted to the dorsal hippocampus at the AP positions between −1.34 and −2.30 from bregma. Image capture involved scanning the full length of the hippocampus at 40× magnification across 5 dorsal hippocampal sections. Each channel was acquired in sequential capture mode so as to excite only the target fluorophore and prevent emission spectrum overlap.

#### Cell quantification

Cell counts were performed using a semiautomated custom-designed macro (ImageJ, National Institutes of Health). Briefly, background subtraction was applied to remove background noise. Images were then converted to binary, and thresholding was used to isolate stained cells. Finally, the Analyze Particles tool was used to quantify the number of positively labeled cells based on a minimum particle size of 16 μm^2^. For colabeled cell quantification, each channel was first examined individually and cells that had signal above threshold were identified and counted. The individual channels were then digitally merged to form a composite image, and the number of colabeled cells was quantified by an experimenter blind to the experimental conditions. To determine DAPI^+^ cell estimates, the volume of the counted area was multiplied by the density of DAPI^+^ cells, as determined by manual counts performed on images taken from 10 animals. The level of reactivation, relative to chance, was calculated by dividing the reactivation rate (((tdTomato^+^/c-fos^+^)/tdTomato^+^) × 100) by the chance level of overlap ((tdTomato^+^/DAPI^+^) × (c-fos^+^/DAPI^+^) × 100).

#### Statistics

Data were analyzed using Student's *t* tests, one-way or two-way ANOVAs, where appropriate. Significant ANOVAs were followed by Tukey *post hoc* tests for multiple comparisons. Statistical significance was defined by α = 0.05 for all analyses. Data were analyzed using Prism version 8 (GraphPad).

## Results

### PSI mediates memory precision

**Figure 1.**
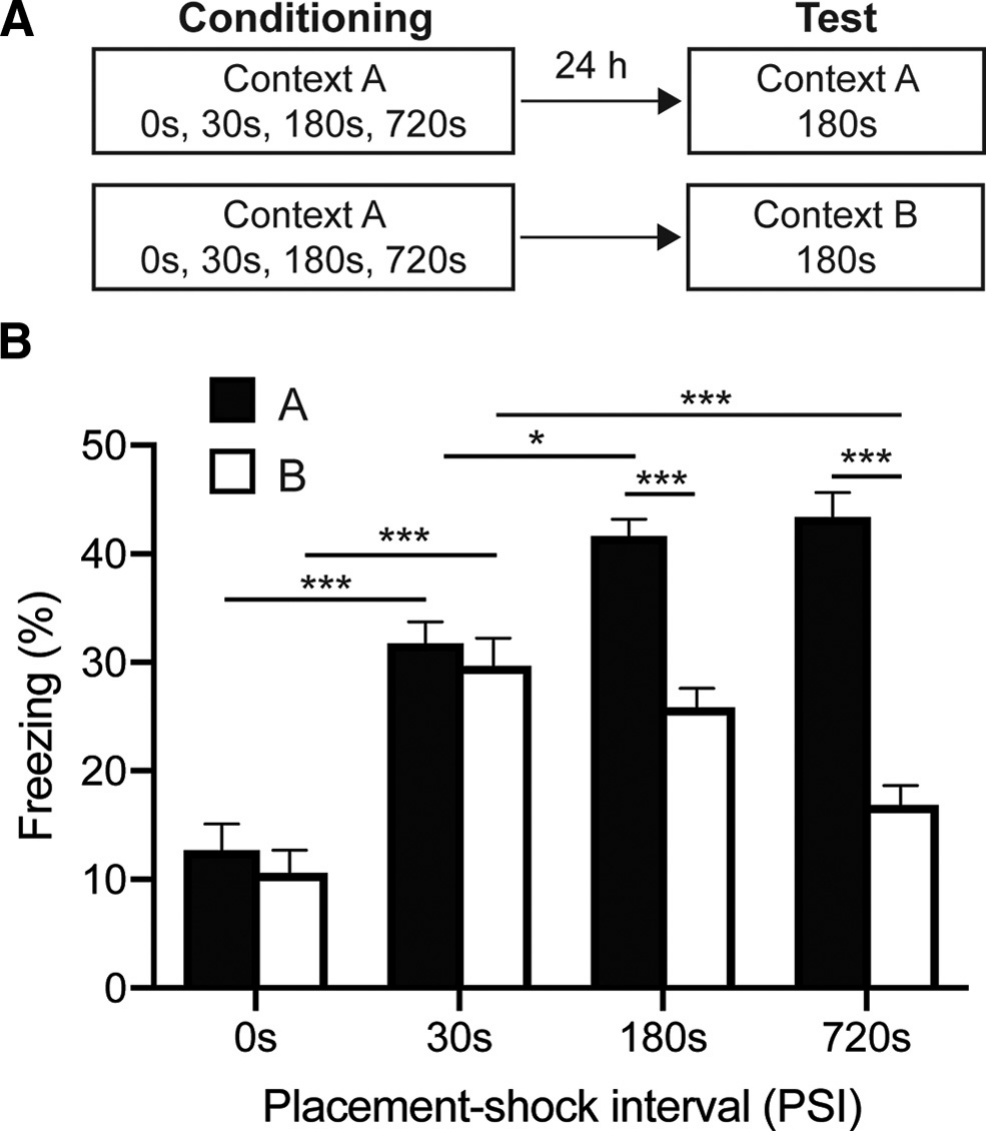
PSI mediates conditional freezing and context discrimination. ***A***, Mice (*n* = 12/group) were placed in a novel context (Context A) for 0, 30, 180, or 720 s before footshock. Twenty-four hours later, mice were tested for 3 min without shock in either Context A or a similar context (Context B). ***B***, Freezing levels in Context A increased with PSI and reached plateau at ∼180 s PSI. Discrimination improved with PSI. At least 180 s in the context before shock was necessary to produce clear discrimination. Data are mean ± SE. Significant differences: *p < 0.05; ***p < 0.001.

We first aimed to establish the relationship between PSI and memory precision. Memory precision was assessed by examining the ability of the mice to discriminate between the conditioning context and a similar context that had never been paired with shock. Mice underwent contextual fear conditioning in Context A at 0 s (immediate shock), or 30, 180, or 720 s PSI (Fig. 1*A*). Twenty-four hours later, mice were returned to either the conditioning context (Context A) or a similar context (Context B) for 3 min without shock. A 3 min test period was selected as we previously found that freezing across 0-720 s PSIs was greatest during the first 3 min of test and was not related to the timing of shock (Leake et al., 2017; Zinn et al., 2020).

Consistent with previous findings (Zinn et al., 2020), both conditional freezing and discrimination increased as a function of PSI, with longer PSIs producing stronger freezing that was preferentially expressed in the shock context (Fig. 1*B*; two-way ANOVA of PSI × context, PSI, *F*_(3,88)_ = 16.45, *p* < 0.001; Context, *F*_(1,88)_ = 47.46, *p* < 0.001; PSI × Context interaction, *F*_(3,88)_ = 63.20, *p* < 0.001). Fear in Context A was initially low, but increased rapidly, reaching plateau at ∼180 s PSI. Fear in Context B peaked at the 30 s PSI and decreased as the PSI lengthened, with significantly less freezing observed at the 720 s PSI compared with the 30 s PSI (*p* < 0.001). Freezing in Context B at the 720 s PSI did not differ significantly from the 0s PSI (*p* > 0.05), indicating that fear was close to baseline. Fear was generalized at 0 and 30 s PSIs and did not differ statistically between contexts (*p* > 0.05 for both comparisons). In contrast, mice conditioned at PSIs of 180 s or longer displayed markedly more fear in Context A than those tested in Context B (*p* < 0.001 for all comparisons). This indicates that PSI mediates context fear memory precision, with longer PSIs producing better context discrimination.

### PSI mediates neural ensemble activity after learning

**Figure 2.**
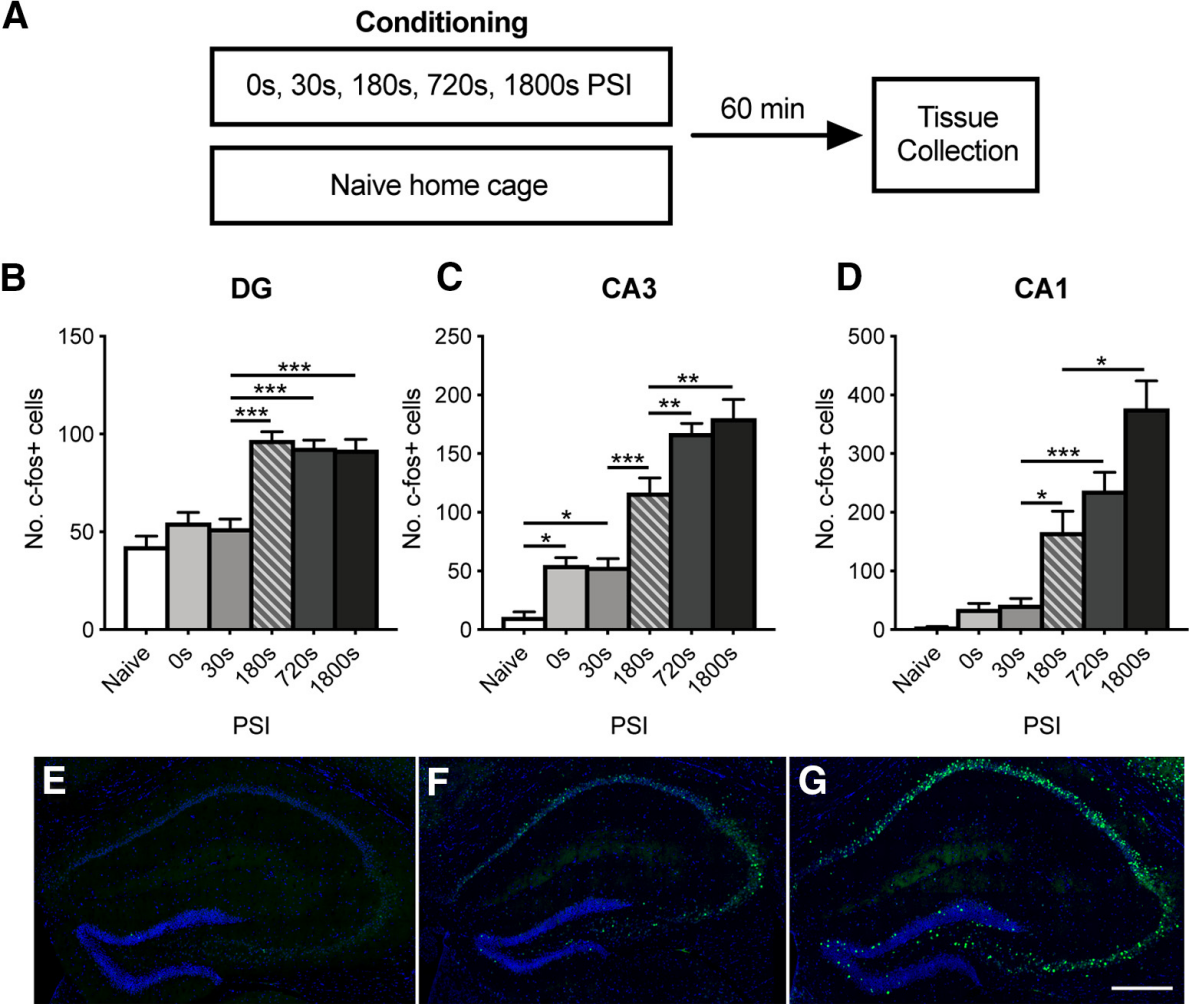
PSI regulates the number of c-fos-expressing cells in the dorsal hippocampus. ***A***, Experimental design. Mice (*n* = 6-8/group) were placed in a novel context for up to 1800 s before footshock. Mice were perfused 60 min after conditioning, and tissue was probed for c-fos expression using IHC. Tissue was also collected from naive home cage controls. The number of c-fos^+^ cells increased as a function of PSI in the DG (***B***), CA3 (***C***), and CA1 (***D***) hippocampal regions. ***E–G***, Representative images of the dorsal hippocampus showing c-fos^+^ cells (green) and DAPI (blue) for the naive (***E***), 30 s PSI (***F***), and 1800 s PSI (***G***) conditions. Scale bar, 20 µm. Data are mean ± SE. Significant differences: *p < 0.05; **p < 0.001; ***p < 0.001.

Next, we assessed cellular alterations associated with the changes in memory precision across PSI. In previous work (Zinn et al., 2020), we proposed that the improvements in memory precision were because of animals acquiring more contextual information as the PSI lengthened. If so, we predicted that longer PSIs would result in the activation of more cells, as these would be required to store additional contextual information within the newly formed neural representation. To test this possibility, mice were conditioned at a 0 s (immediate shock), or 30, 180, 720, or 1800 s PSI (Fig. 2*A*). Sixty minutes after conditioning, mice were perfused and brains were removed for immunohistochemical analysis of c-fos. Tissue was also collected from naive home cage animals, which served as a baseline control.

Consistent with our predictions and recent findings (Colon and Poulos, 2020), c-fos expression increased across PSI within each of the hippocampal subregions (Fig. 2*B-G*; one-way ANOVA of c-fos^+^ cells: DG; *F*_(5,41)_ = 5.187, *p* < 0.001, CA3; *F*_(5,41)_ = 11.45, *p* < 0.001, CA1; *F*_(5,41)_ = 8.288, *p* < 0.001). Within the DG, the number of c-fos^+^ cells was low in control groups (0 s and naive) and increased significantly at 180 s PSI (*p* < 0.001). Extending the PSI up to 1800 s did not result in any further increases in the number of c-fos^+^ cells (*p* > 0.05 for all comparisons), indicating that activity reached plateau with 180 s of experience. In contrast, in the CA3, the number of c-fos^+^ cells increased from baseline at 0 s PSI (*p* < 0.05) and only reached plateau at 720 s PSI (180 s PSI vs 720 s PSI, *p* < 0.01). Finally, in the CA1, the number of c-fos^+^ cells was significantly higher than controls at 180 s PSI (*p* < 0.05), and reached plateau only at 1800 s PSI (720 s vs 1800 s PSI, *p* < 0.05). These results demonstrate that extended time in the context before shock results in the activation of more hippocampal cells, and that this increase is larger in the CA regions compared with the DG. This is consistent with our hypothesis that animals represent more contextual information as the session progresses, enabling more precise discrimination.

### PSI does not mediate the pattern of neuronal ensemble activity at test

Our next goal was to examine whether the duration of initial context exposure also influences the expression of c-fos activation after recall. It is widely believed that recall involves reactivation of the memory trace formed during initial learning (Reijmers et al., 2007; Liu et al., 2012). Given that, as suggested by the previous result (compare Fig. 2), the memory trace likely involves fewer cells at shorter PSIs, we hypothesized that the maximal number of cells activated at recall would be lower at shorter PSIs. Additionally, we proposed that the original memory trace could take longer to be fully activated at shorter PSIs. This might occur for two reasons. First, given that fewer contextual features are incorporated into the memory, more time might be needed for them to be resampled to reactivate the original representation. Second, poor learning may cause weaker synaptic connectivity between engram cells, reducing the likelihood of full memory reactivation through CA3 collateral activity.

**Figure 3.**
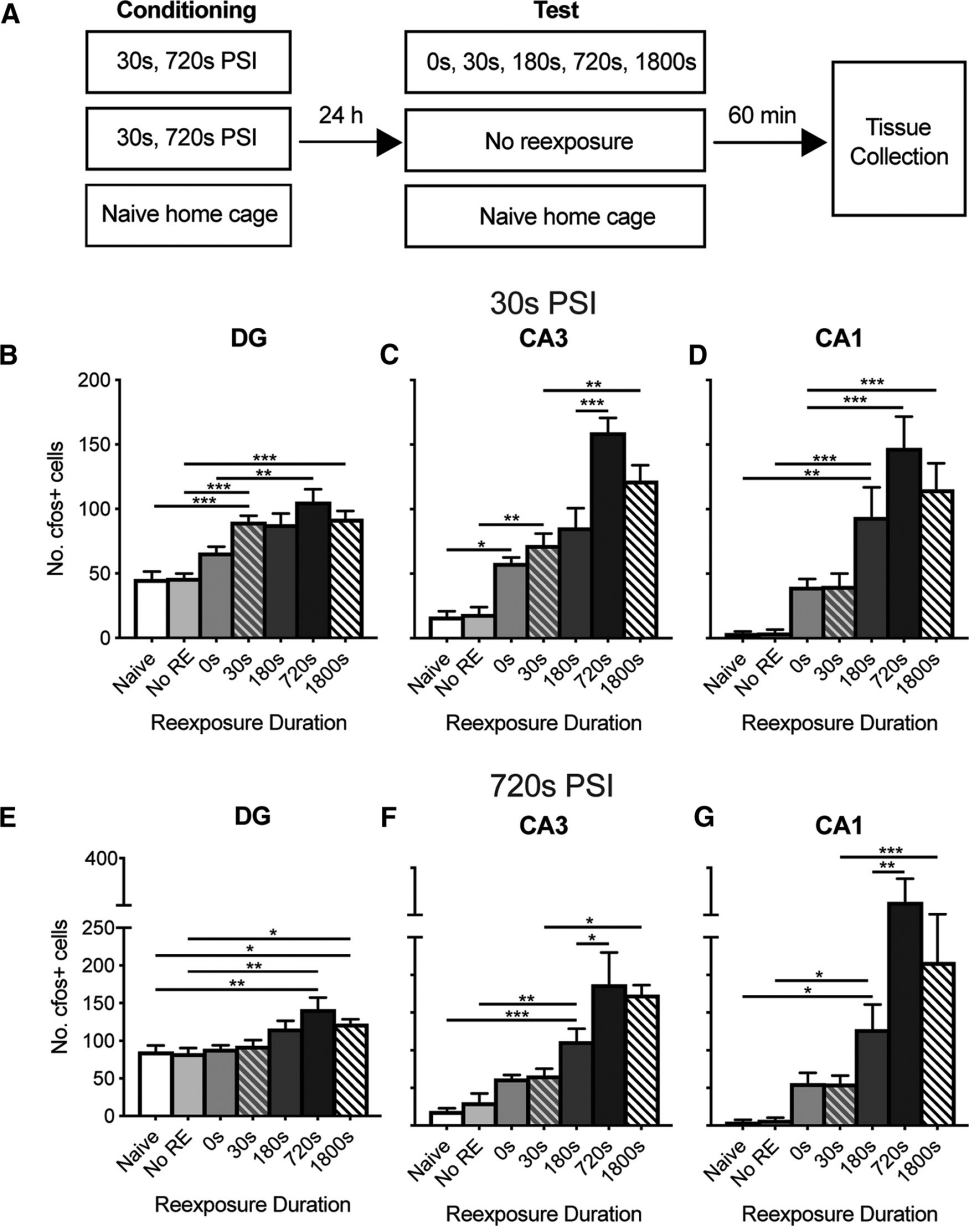
Time course of c-fos activation following retrieval of a 30 or 720 s PSI memory. ***A***, Experimental design. Mice (*n* = 7 or 8/group) were placed in a novel context for 30 or 720 s before footshock. Twenty-four hours later, mice were returned to the conditioning context for up to 1800 s before being returned to their home cage. A separate group of mice was conditioned but not reexposed to the context. Mice were perfused 60 min after conditioning, and tissue was probed for c-fos expression using IHC. Tissue was also collected from naive home cage controls. The number of c-fos^+^ cells increased as a function of reexposure duration in the DG (***D***,***E***), CA3 (***C***,***F***), and CA1 (***D***,***G***) hippocampal subregions at both the 30 s PSI (***B–D***) and the 720 s PSI (***E–G***). Data are mean ± SE. Significant differences: *p < 0.05; **p < 0.001; ***p < 0.001.

To begin testing these possibilities, we assessed the pattern of c-fos activation at each PSI separately. Mice were conditioned at either a training PSI of 30 or 720 s and then reexposed to the context for various periods of time on the following day (Fig. 3*A*). These PSIs were selected as they produced imprecise (30 s PSI) and precise (720 s PSI) memories as established in Figure 1. Sixty minutes after context reexposure, mice were perfused and brains were removed for immunohistochemical analysis of c-fos. As a control, tissue was also collected from naive untreated animals and animals that were conditioned but not reexposed to the conditioning context. The number of c-fos^+^ cells increased with reexposure duration in all hippocampal subregions and at both PSIs (Fig. 3*B-G*; 30 s PSI, DG *F*_(6,48)_ = 12.1, *p* < 0.001, CA3 *F*_(6,48)_ = 29.33, *p* < 0001, CA1 *F*_(6,48)_ = 12.48, *p* < 0.001, 720 s PSI, DG *F*_(6,42)_ = 6.355, *p* < 0.001, CA3 *F*_(6,42)_ = 21.46, *p* < 0.001, CA1 *F*_(6,42)_ = 14.68, *p* < 0.001). Contrary to our predictions, the maximum number of activated cells was reached at the same reexposure duration at both PSIs, with the DG reaching plateau after 180 s (Fig. 3*B*,*E*) and the CA3 and CA1 reaching maximum after 720 s (Fig. 3*C*,*D*,*F*,*G*). Further, both PSIs exhibited a slight decrease in c-fos^+^ cells at the 1800 s time point, although this difference was not statistically significant compared with the 720 s time point (Fig. 3*B-G*; *p* > 0.05 for all comparison). Although this difference was not statistically significant at either PSI, it could potentially reflect degradation of some of the c-fos protein generated at the start of the session because of the longer time period before tissue collection. Together, these data suggest that PSI does not influence the rate of cellular activation at recall.

### PSI does not mediate the level of neuronal ensemble activity at test

In the previous experiment (compare Fig. 3), retrieval of a memory formed at either 30 or 720 s PSI appeared to produce a similar pattern of c-fos induction, with fewer cells activated at the start of the session and maximal cellular activation after 180-720 s of context exposure. This pattern also appeared similar to that observed after initial conditioning (compare Fig. 2). This led us to hypothesize that the absolute number of cells active after a given session reflects the duration of the current session, independent of previous learning. Given that each of these datasets were collected as part of separate experiments, they could not be compared directly. We therefore sought to confirm our hypothesis by performing a new experiment in which we directly compared the number of cells activated after conditioning, with those active after recall sessions of equivalent durations, following conditioning at different PSIs.

**Figure 4.**
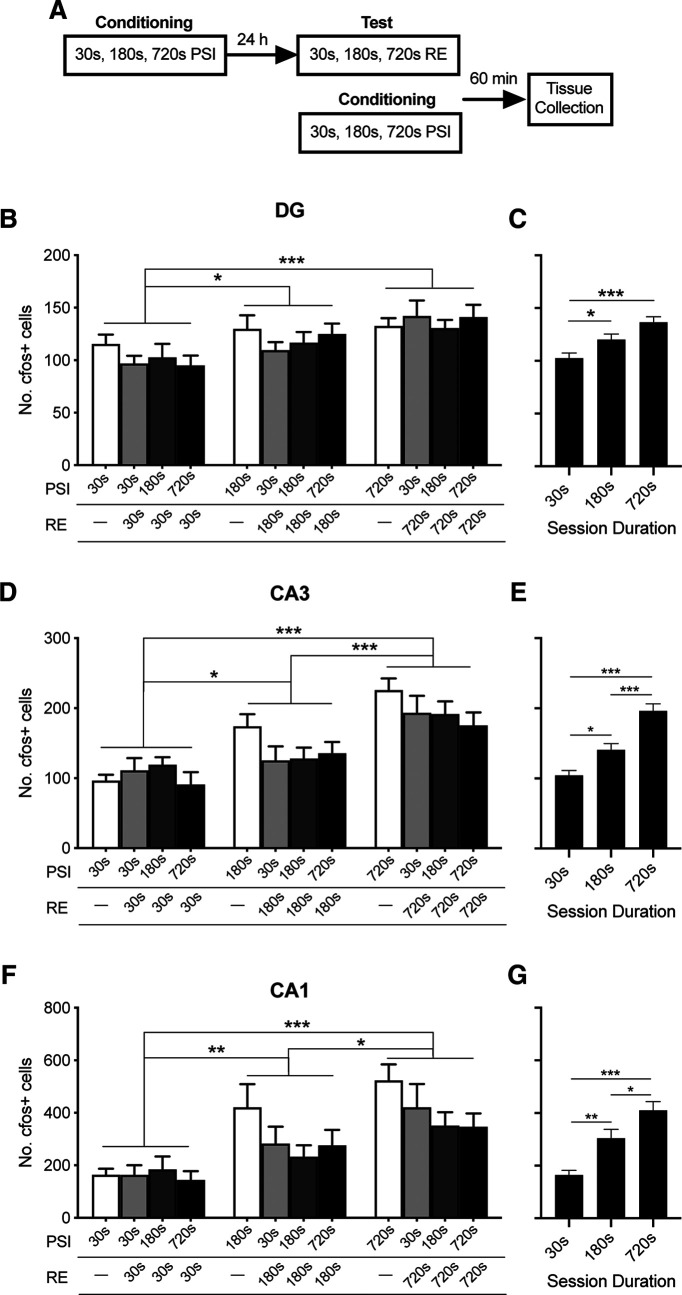
c-fos levels at test are regulated by current session duration rather than learning history. ***A***, Experimental design. Mice (*n* = 8/group) were placed in the context for 30, 180, or 720 s before footshock. Twenty-four hours later, mice were reexposed to the context for 30, 180, or 720 s. Sixty minutes after reexposure, animals were perfused and tissue was collected for immunohistochemical analysis of c-fos. Tissue was also collected from a separate group of mice after conditioning at 30, 180, or 720 s PSI. The number of c-fos^+^ cells increased as a function of reexposure duration in the DG (***B***), CA3 (***D***), and CA1 (***F***). ***C***, ***E***, ***G***, Data from ***B***, ***D***, ***F***, collapsed across learning history for ease of comparison. There was no significant effect of previous PSI on the number of c-fos^+^ cells. Data were collected from four replications with similar results. Data are mean ± SE. Significant differences: *p < 0.05; **p < 0.001; ***p < 0.001.

To achieve this, on day 1, mice were conditioned at 30, 180, or 720 s PSI (Fig. 4*A*). The following day, they were returned to the conditioning context for 30, 180, or 720 s. A separate group of mice received no conditioning on day 1 and were conditioned on day 2 at 30, 180, or 720 s PSI. All mice were perfused 60 min later, and brains were collected for immunohistochemical analysis of c-fos.

In order to compare the number of c-fos^+^ cells across the relevant groups, we performed two-way ANOVAs comparing the effect of PSI duration on day 1 with the duration of the reexposure session on day 2. In each case, we compared across equivalent context durations, such that tissue collected immediately after the 30 s PSI was compared with the 30 s reexposure groups, at various PSIs, and so forth for the other conditions. Consistent with our previous findings (Fig. 3), the number of c-fos^+^ cells increased across test session duration within each of the hippocampal subregions (Fig. 4*B-D*; DG, *F*_(2,84)_ = 10.88, *p* < 0.001; CA3, *F*_(2,84)_ = 29.01, *p* < 0.001; CA1, *F*_(2,84)_ = 18.66, *p* < 0.001). However, the number of c-fos^+^ cells did not differ significantly across PSIs (DG, *F*_(3,84)_ = 0.570, *p* > 0.05; CA3, *F*_(3,84)_ = 1.776, *p* > 0.05; CA1, *F*_(3,84)_ = 2.621, *p* > 0.05). Critically, there was no significant interaction, indicating that c-fos activity after context reexposure did not differ as a function of initial PSI (DG, *F*_(6,84)_ = 0.652, *p* > 0.05; CA3, *F*_(6,84)_ = 1.012, *p* > 0.05; CA1, *F*_(6,84)_ = 0.782, *p* > 0.05). Therefore, despite some groups having two sessions in the context, and others having just one, the size of c-fos-expressing cell population was unchanged. These results together suggest that the total number of c-fos^+^ cells is influenced by the duration of the session immediately preceding c-fos assessment and not by the extent of previous contextual experience.

### Memory precision corresponds with reactivation of the DG ensemble involved in learning

Experiment 5 indicated that differences in the number of c-fos^+^ neurons between PSIs were not apparent when assessing the total population of c-fos^+^ cells active at retrieval. However, previous research has shown that the degree of overlap between IEG^+^ cells activated after conditioning and testing is remarkably low, suggesting that only a fraction of the original cell population becomes incorporated into the memory (Liu et al., 2012; Tayler et al., 2013). Our next aim was therefore to test the hypothesis that longer PSIs, which produce more precise memories, are associated with increased reactivation of cellular ensembles that encode the feared context.

In order to test this possibility, we used the *TRAP2* mouse line to permanently label neurons active after conditioning at either a 30 or 720 s PSI (Allen et al., 2017; DeNardo et al., 2019). In these mice, *c-fos* expression drives the integration of tamoxifen-inducible Cre recombinanse (CreER^T2^). When a neuron is active in the presence of tamoxifen, or its metabolite 4-OHT, CreER^T2^ translocates to the nucleus to initiate recombination and permanent expression of an effector gene. In this case, *TRAP2* mice were crossed with a tdTomato reporter line (Ai14) to produce double transgenic *TRAP2:Ai14* mice.

**Figure 5.**
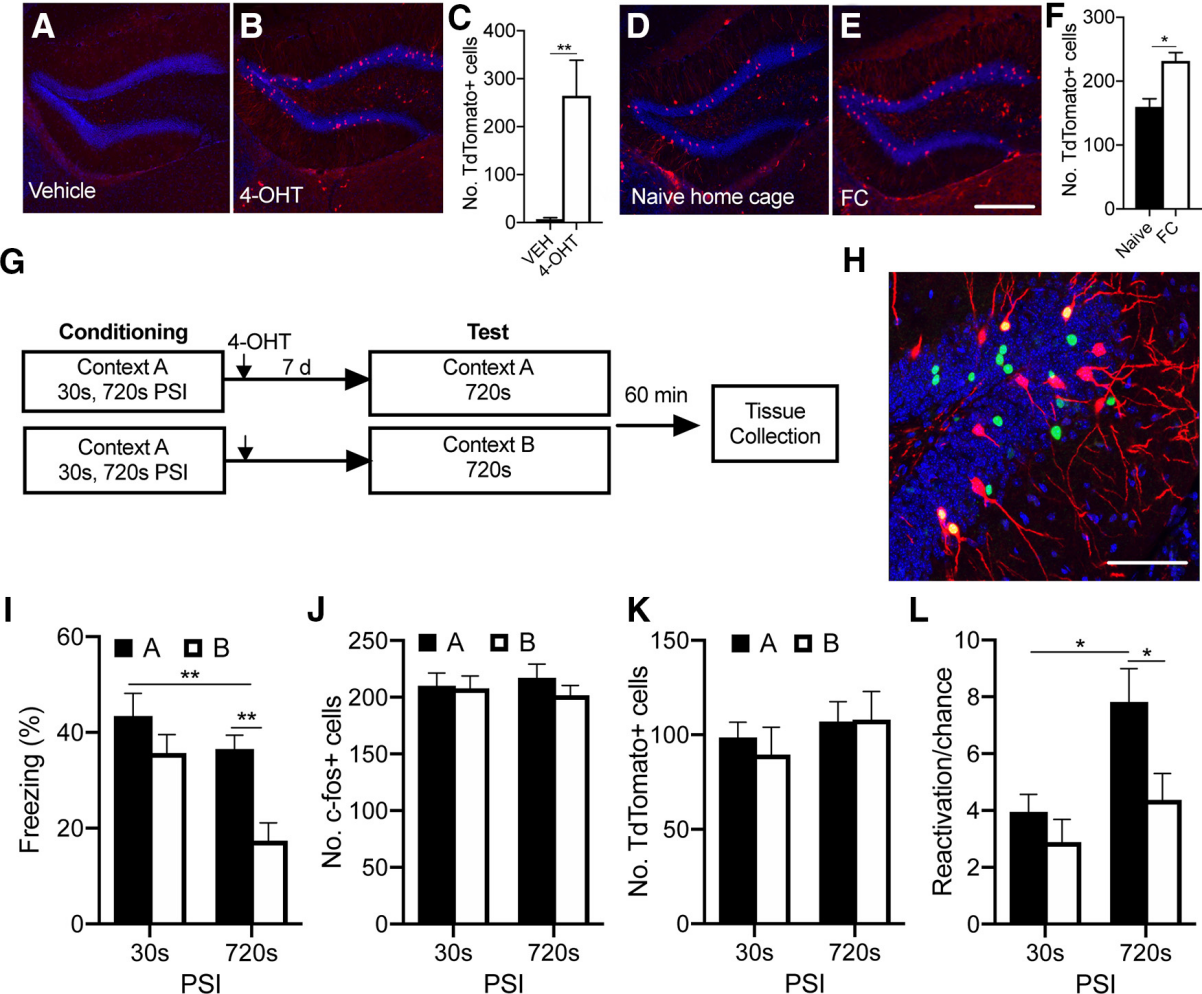
PSI regulates cellular reactivation in the DG. ***A–C***, Validation of tdTomato expression in the DG in response to 4-OHT. ***C***, tdTomato expression was almost entirely absent in vehicle-injected animals. ***D***–***F***, tdTomato is more highly expressed in fear-conditioned animals. Scale bar, 250 μm. ***G***, Mice (*n* = 14/group) were placed in Context A for 30 or 720 s before footshock. Seven days later, mice were exposed to either Context A or a similar context (Context B) for 720 s without shock. Mice were perfused 60 min after testing, and tissue was probed for tdTomato and c-fos expression using IHC. ***I***, Mice were able to differentiate between similar contexts at the 720 s PSI, but not the 30 s PSI. ***J***, ***K***, There were no significant differences in the number of c-fos^+^ or tdTomato^+^ cells across conditions. ***L***, At the 720 s PSI, cells that were active during learning were preferentially reactivated in Context A, compared with Context B. At the 30 s PSI, reactivation was low and did not differ significantly between contexts. ***H***, Representative fluorescent confocal images showing cells labeled with tdTomato (red), c-fos (green), DAPI (blue), and colabeled tdTomoto/c-fos^+^ cells (yellow). Scale bar, 75 μm. Data are mean ± SE. Significant differences: *p < 0.05; **p < 0.001.

We first validated that the tdTomato reporter in the hippocampus of *TRAP2* mice could be regulated by 4-OHT. Mice underwent context fear conditioning and were subsequently injected with either 4-OHT or vehicle. We found that tdTomato expression was almost entirely absent in the hippocampus of vehicle animals but was present in 4-OHT-injected animals (Fig. 5*A-C*). It was notable that the efficiency of tagging in the CA3 and CA1 hippocampal subregions was low, as has been reported for a number of other IEG-driven transgenic mice (Deng et al., 2013; Cazzulino et al., 2016). As a result, all further analyses with the *TRAP:Ai14* mice were restricted to the DG.

Next, we examined whether tdTomato was expressed in an activity-dependent manner in the DG. Mice underwent fear conditioning or remained in their home cage and were then injected with 4-OHT. While tdTomato^+^ cells could be detected in home cage animals, there were significantly more tdTomato^+^ cells in those animals that underwent fear conditioning (Fig. 5*D-F*; *t*_(5)_ = 3.214, *p* > 0.05). This confirms the utility of this mouse line for examining neural activity associated with fear learning and memory.

In order to test the effect of PSI on cellular reactivation in the DG, mice underwent context fear conditioning at either a 30 or 720 s PSI and were then returned to either the conditioning context (Context A) or a similar context (Context B) for 720 s without shock. In contrast to our earlier results (compare Fig. 1), the level of freezing at the 30 s PSI did not differ significantly from that observed at the 720 s PSI (*p* > 0.05). This may reflect mouse strain-dependent differences in the exact time point at which conditioning reaches plateau, as has been previously observed (Fanselow, 1986). Nonetheless, as observed previously, animals conditioned at a 720 s PSI displayed significantly higher freezing in Context A than B, whereas those conditioned at a 30 s PSI showed similar levels of freezing in both contexts. This reproduces the effect of PSI on memory precision in the transgenic mice (Fig. 5*I*; effect of PSI *F*_(1,52)_ = 10.68, *p* < 0.01, effect of context *F*_(1,52)_ = 12.13, *p* < 0.01, PSI × context interaction, *F*_(1,52)_ = 2.19).

All mice were perfused 60 min after the test session to assess reactivation of learning-tagged cells in either Context A or Context B. There were no differences in the number of c-fos^+^ cells across groups, as would be expected given that the duration of the test session was the same across all conditions (Fig. 5*J*; effect of PSI, *F*_(1,52)_ = 0.6, *p* > 0.05, effect of context, *F*_(1,52)_ < 0.0, *p* > 0.05, interaction, *F*_(1,52)_ = 0.37, *p* > 0.05). In contrast to our previous c-fos results (Fig. 2), there was no significant difference in the number of tdTomato^+^ cells across groups (Fig. 5*K*; effect of PSI, *F*_(1,52)_ = 0.16, *p* > 0.05, effect of context, *F*_(1,52)_ = 1.2, *p* > 0.05, interaction, *F*_(1,52)_ = 0.12, *p* > 0.05). However, we previously observed that the difference in cellular activity between the 30 and 720 s PSI was much smaller in the DG compared with the CA3 and CA1 subregions (Fig. 2). This smaller effect size, combined with a longer 4-OHT-mediated labeling window (6 h), likely obscured the difference that was previously observed.

Nonetheless, in line with our predictions, there was greater reactivation in Context A compared with Context B at the 720 s PSI (*p* < 0.05), but not the 30 s PSI (*p* > 0.05). Interestingly, this difference did not arise because of less reactivation in Context B, but rather because of more reactivation in Context A at the 720 s PSI compared with the 30 s PSI (Fig. 5*L*; effect of PSI, *F*_(1,52)_ = 8.91, *p* < 0.01, effect of context, *F*_(1,52)_ = 6.32, *p* < 0.05, interaction, *F*_(1,52)_ = 1.76, Tukey *post hoc* comparison, 30 s PSI A vs 720 s PSI A, *p* < 0.05, 30 s PSI B vs 720 s PSI B, *p* > 0.05). These results cannot simply be attributed to the level of conditioning as there was no significant correlation between reactivation rate and freezing levels in the conditioning context (*r* = −0.10, *p* > 0.05).

These findings have three implications. First, the level of reactivation does not simply track the level of freezing; otherwise, reactivation would have been similar across PSIs in Context A. Second, the 720 s PSI produces a larger engram, consistent with the encoding of more contextual information. Finally, the degree of discrimination across different PSIs tracks the proportion, rather than the absolute number, of engram cells reactivated, with a lower proportion reactivated in different contexts corresponding with discrimination.

## Discussion

**Figure 6.**
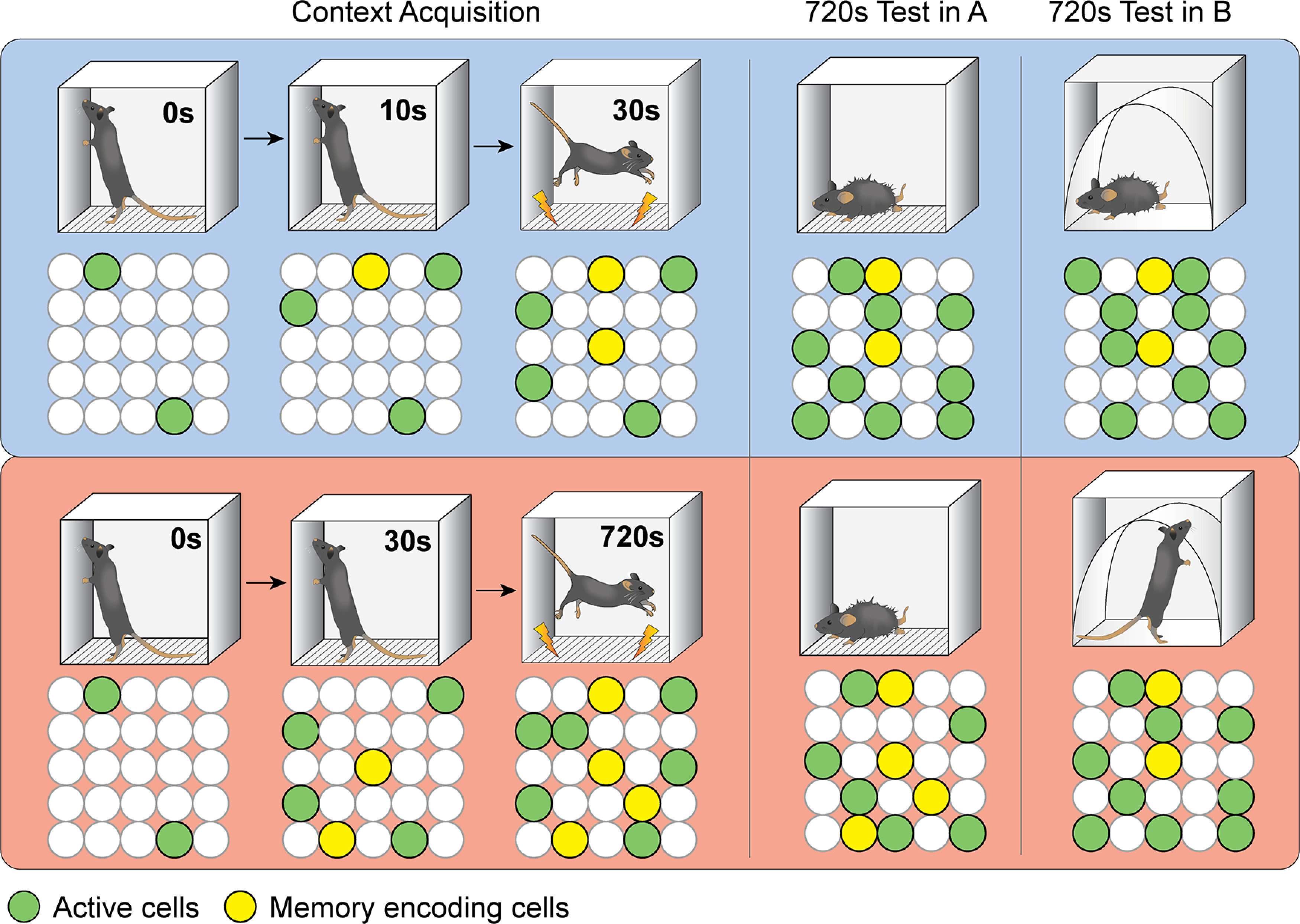
A model for ensemble activity in the DG during the formation and retrieval of memories acquired at different PSIs. Context exploration evokes activity in a population of cells in the DG (yellow and green cells). A subpopulation of these cells will go on to form the neural trace of the memory (yellow cells). As the PSI increases, animals have more time to sample and encode the features of the context, resulting in an increase in both the number of active cells and the number of memory-encoding cells. During testing, mice are exposed to either the same context (A) or a slightly different context (B) for an extended period of time (12 min). When tested in Context A, this time period is sufficient for animals to reencounter the original contextual features, resulting in reactivation of the memory trace and producing freezing behavior. On the other hand, at the 30s PSI (upper panel, blue background), fewer cells encode the memory during learning; therefore, fewer cells are reactivated at test. Because less information was encoded, animals have fewer unique details with which to discriminate between contexts. When tested in Context B, the shared features lead to reactivation of the original memory trace, and thus to generalized freezing. On the other hand, at the 720s PSI (lower panel, red background), animals encode features increasingly unique to context A, thus allowing them to separate the neuronal pattern in and thus discriminate in context B.

This study investigated the neural basis of learning-dependent changes in memory precision. We found that the total number of c-fos-expressing cells increased to plateau with the duration of the current session, regardless of learning history. In contrast, the subset of cells that were active during learning and reactivated at test corresponded with the degree of initial learning and subsequent discrimination. These results indicate that the number of reactivated cells, rather than the total number of active cells, is a more appropriate signature of the neural engram underlying the memory. Our interpretation of these findings, as schematized in Figure 6, is that the extent of initial learning determines the size of the reactivated engram population, which controls the degree of discrimination. Thus, poorer learning produces smaller engrams that impair memory separation and behavioral discrimination.

### More contextual information is encoded by more cells

We found that the number of reactivated cells seen after recall was greater at the 720 s PSI compared with the 30 s PSI. This finding cannot simply be attributed to the level of fear expressed, as fear of Context A was equivalent between PSIs and there was no correlation between reactivated cell numbers and freezing. Instead, we propose that the double-labeled cells represent the quantity of information acquired, with longer PSIs providing more time to encode contextual information and thus increasing the number of reactivated cells.

This idea would seem to contradict previous research indicating that the neuronal representation of the memory is stereotyped in size and does not differ across experience (Rao-Ruiz et al., 2019). Within the DG, 2%-8% of cells are activated during memory formation, regardless of task valence or the type of conditioning (Liu et al., 2012; Tayler et al., 2013; Redondo et al., 2014). The proportion of these cells that are reactivated at test is also consistent across tasks and experiences, likely because of intrinsic excitability and inhibitory circuits limiting maximal engram size (Tayler et al., 2013; Redondo et al., 2014; Park et al., 2016).

However, such stability need not contradict our findings. It is possible that previous studies used conditions that maximally activated the DG, while missing the variable activity range we observed with shorter intervals. If so, this may mean that, once maximally activated, the size of the engram remains stable. However, before this, engram size can differ, reflecting differences in memory content that can have significant implications for cognition and behavior.

### A larger ensemble supports better discrimination

According to conventional theories of pattern separation, the DG maintains memory specificity by encoding memories in sparse nonorthogonal representations (McNaughton and Morris, 1987; Treves and Rolls, 1992; O'Reilly and McClelland, 1994). Thus, recruiting more cells would make the memory less precise because it would increase the likelihood that any two memories would be encoded by the same cells. In contrast, recent models propose that more cells are necessary to encode richer experiential information, allowing better discrimination in downstream regions (Aimone et al., 2011).

Here, the number of cells recruited into the memory naturally varied with the duration of the learning period. This provides a unique situation in which to observe how engram size relates to subsequent memory precision. We found that, although freezing in Context B differed between PSIs, the number of reactivated cells was the same. Moreover, the opposite was true in Context A, with freezing being equivalent but the number of reactivated cells being different across PSIs. This indicates that behavioral discrimination is not simply a readout of the absolute number of reactivated cells in a given context. Rather, discrimination corresponds with the proportion of the Context A engram that gets reactivated in Context B. With this in mind, we found that the 720 s PSI produced a larger engram that was less engaged in Context B, corresponding with behavioral discrimination. Conversely, the 30 s PSI produced a smaller engram that was engaged to a similar extent in both contexts, mirroring behavioral generalization. These findings suggest that larger engrams support, rather than hinder, discrimination and that this discrimination is associated with greater cellular reactivation in the conditioning context relative to a different context.

These findings are consistent with the proposed function of the DG in maintaining memory resolution, with the recruitment of more cells during learning supporting better memory specificity, presumably via the encoding of more information into the memory (Aimone et al., 2011). They also agree with studies examining remote memory (Wiltgen et al., 2010) and artificial manipulations of neural inputs to the hippocampus (Xu and Sudhof, 2013), which found that better discrimination was associated with more IEG-expressing hippocampal cells. Nonetheless, our findings do not entirely preclude the idea that overall sparseness is required for memory precision. The time-dependent increases in cellular activity we observed were small, such that even at the 720 s PSI, only ∼2% of the total DG population was activated. Thus, activity was still sparse even at the longer time intervals. We suggest that, while very large increases in cellular activity may cause interference between related memories and thus impair memory precision (Ruediger et al., 2011; Basu et al., 2016), smaller physiological increases are beneficial as they allow more contextual information to be encoded into the memory.

### Total c-fos activity reflects time-dependent hippocampal processing

Memory precision corresponded with cellular reactivation in a subset of c-fos-expressing cells. In contrast, the total number of c-fos-expressing cells was unrelated to previous learning history and instead more closely reflected the duration of the current session. However, total c-fos expression did not appear to track time itself, as its increase was not linear and reached plateau after ∼12 min in the context. This raises the question as to what factors trigger c-fos expression during the session.

Research has shown that c-fos-expressing cells are required for memory encoding and retrieval, as inactivating the entire population of cells that were active during learning produces amnesia (Tanaka et al., 2014; Matsuo, 2015). However, our experiments, and those of others, indicate that only a small proportion of c-fos-expressing cells are reactivated at test (Liu et al., 2012; Tayler et al., 2013). This suggests that the majority of c-fos^+^ cells are either not involved in memory storage, or their involvement cannot be detected by the methods assessed.

Additionally, we found that many cells expressed c-fos during testing that were not tagged during conditioning. This raises the possibility that these cells might be engaged in new learning or updating processes. Indeed, research has shown that c-fos-expressing cells are involved in post-retrieval processes, including extinction and memory updating (Mamiya et al., 2009; Ryan et al., 2015; Bernier et al., 2017; Lacagnina et al., 2019). However, previous research (Zinn et al., 2020) indicates that extinction and updating are differentially regulated by PSI, with retrieval of shorter PSI memories supporting updating and retrieval of longer PSI memories supporting extinction. Despite these differences, we here observed the same increase in total c-fos expression across test sessions, regardless of PSIs. Thus, while these processes might be occurring in a subset of reactivated cells, the pattern of total c-fos expression cannot be accounted for by extinction or updating alone.

Rather than reflecting any single cognitive process, we suggest that total c-fos expression is indicative of the degree to which the hippocampus has been stimulated during the current session. Accordingly, stimulation would decrease across a session as the context becomes more familiar and would reoccur on reentry into the context. This account accords with the long-held view that IEG expression captures the activity of place cells, which emerge rapidly but take time to develop over the course of environmental exposure (Guzowski et al., 1999; Frank et al., 2004; Vazdarjanova and Guzowski, 2004). It is also consistent with recent demonstrations that c-fos-expressing cells develop place fields that correspond with contextual identity (Tanaka et al., 2018). Further research is needed to verify this hypothesis and clarify the cognitive processes regulating c-fos induction in response to ongoing experience.

In conclusion, this work supports the idea that fear generalization depends not only on the similarity of environments, but also critically on the extent of initial learning (Kiernan and Westbrook, 1993; Westbrook et al., 1994). Moreover, it identifies a potential neural mechanism for these effects, with better learning resulting in recruitment of larger hippocampal ensembles to encode detail-rich contextual information that can aid discrimination.

While memory generalization can be beneficial, overgeneralization of fear is maladaptive and a hallmark of psychiatric disorders, including post-traumatic stress disorder (Dunsmoor and Paz, 2015). Our findings, and those of others, suggest that insufficient contextual processing during trauma could be one mechanism through which generalized fear arises (Jacobs and Nadel, 1985; Brewin et al., 2010; Brewin, 2014; Zinn et al., 2020). Extending on this, recent research indicates that generalization induced by insufficient contextual learning can be ameliorated by further context exposure, allowing memories to be updated with additional information (Zinn et al., 2020). Future studies could investigate whether further context learning reduces generalization by recruiting more cells into the memory, which subsequently support better separation between hippocampal representations.
